# Invasive Listeriosis in an Otherwise Immunocompetent Elderly Patient

**DOI:** 10.7759/cureus.111314

**Published:** 2026-06-22

**Authors:** Carolyn K Kan, Sarah Chan, Jetrina Maque, Jacqueline Cervantes, Christina W Chung

**Affiliations:** 1 Internal Medicine, West Los Angeles Veterans Affairs, Los Angeles, USA; 2 Internal Medicine, UCLA (University of California, Los Angeles) Medical Center, Santa Monica, USA; 3 Internal Medicine, Los Angeles County and University of Southern California (LAC+USC) Medical Center, Los Angeles, USA

**Keywords:** ampicillin sodium, bacteremia, elderly, invasive listeriosis, listeria, listeriosis, meningitis

## Abstract

*Listeria monocytogenes* is a foodborne, intracellular Gram-positive bacillus that typically causes mild gastrointestinal illness or flu-like symptoms in healthy hosts but can lead to invasive disease, including bacteremia and central nervous system infection. Although classically associated with immunocompromised states, advanced age has emerged as an independent risk factor for invasive listeriosis. We report a case of an otherwise healthy elderly male patient who presented with nonspecific systemic symptoms and was subsequently found to have *Listeria monocytogenes* bacteremia and meningitis. Initial presentation was atypical for bacterial meningitis, contributing to diagnostic uncertainty. Blood cultures and cerebrospinal fluid analysis ultimately confirmed the diagnosis of invasive listeriosis. The patient was treated with targeted antimicrobial therapy, including ampicillin-based treatment, with clinical management guided by the susceptibility of *Listeria* species. This case report highlights the importance of having a high clinical suspicion of invasive listeriosis in the geriatric population given atypical meningitis symptoms, high mortality rate, and need for appropriate and time-sensitive antibiotic coverage.

## Introduction

*Listeria monocytogenes* is a facultative intracellular, Gram-positive bacillus most commonly acquired through ingestion of contaminated food products, including unpasteurized dairy, deli meats, and other ready-to-eat foods. Clinical manifestations range from mild, self-limiting gastrointestinal illness and nonspecific flu-like symptoms such as fever, myalgias, and fatigue to severe invasive disease [1]. When invasive infection occurs, *L. monocytogenes *demonstrates a predilection for the central nervous system (CNS) and bloodstream, leading to clinical syndromes such as bacteremia, meningitis, and meningoencephalitis. In the retrospective cohort described by Corral et al., CNS involvement was observed in 36.6% of cases, compared with 37.1% presenting with isolated bacteremia [2]. Although relatively uncommon in the general population, with an estimated incidence of approximately 0.24 cases per 100,000 individuals annually in the United States, listeriosis remains clinically important due to its severity and high case fatality rate once invasive disease develops [1].

Classically, invasive listeriosis has been described in patients with impaired cell-mediated immunity, including those with malignancy, solid organ transplants, diabetes mellitus, cirrhosis, chronic alcohol abuse, pregnancy, HIV/AIDS, and immunosuppressive medication exposure [3]. However, advancing age has increasingly been recognized as an independent risk factor for infection, even in the absence of traditional immunocompromising conditions. Epidemiologic data demonstrate a steep age-related gradient in disease incidence, with individuals in the oldest age groups experiencing markedly increased risk compared to younger adults [4]. This vulnerability is largely attributed to immunosenescence, characterized by progressive dysfunction of T-cell-mediated immunity, impaired macrophage activity, and altered cytokine signaling, all of which reduce host defense against intracellular pathogens such as *L. monocytogenes* [5]. Clinically, elderly patients with *L. monocytogenes *meningitis are more likely to have atypical presentations, thereby complicating early recognition. Additionally, *L. monocytogenes*’ unique penicillin-binding proteins (PBPs) makes them intrinsically resistant to cephalosporins, and therefore empiric meningitis regimens that do not include anti-listerial coverage may result in delayed effective therapy, particularly in older adults and other at-risk populations [6].

This case highlights simultaneous *L. monocytogenes* bacteremia and meningitis in an elderly patient without known immunocompromising conditions, underscoring the need to maintain a high index of suspicion for invasive listeriosis in the geriatric population. Given the substantial morbidity and mortality associated with delayed diagnosis and inadequate antibiotic therapy, early recognition and initiation of targeted antimicrobial treatment are essential to improving outcomes. This report further supports that age alone can be a significant risk factor for invasive *L. monocytogenes *infection, even in otherwise immunocompetent hosts.

## Case presentation

An 88-year-old Japanese-speaking male with a prior history of aortic stenosis and gout was brought in by family after a ground-level fall and confusion. Further history was provided by the wife due to the patient’s encephalopathy. Beyond frequent falls over the past few days, patient appeared to be at his baseline mentation (alert and oriented to person, place, and time) and independent with his activities of daily living (ADLs) until the day of presentation when the patient had two ground-level falls with subsequent acute confusion. Wife denied head strike, fevers, chills, dyspnea, cough, chest pain, headaches, rashes, abdominal pain, nausea, vomiting, or recent sick contacts. His only medication was allopurinol, and he had never been on blood thinners previously.

In the emergency department, his blood pressure was 185/105, with a heart rate of 130 beats per minute. His temperature was 40.1°C, and he was oxygenating at 95% breathing ambient air. Physical exam was remarkable for diaphoresis, right auricular hematoma, and laceration to the left mastoid and posterior head region secondary to his fall. Neurological exam revealed the patient to be alert and oriented to person only. Glasgow Coma Scale (GCS) score was deemed to be 14 (4/4/5). He was moving all extremities symmetrically, with no nystagmus, with strength and sensation grossly intact in all extremities. The patient did not demonstrate signs of elevated intracranial pressure, such as abnormal ocular motility, dilated nonreactive pupils, or gaze palsies. Initial bloodwork revealed leukocytosis, anemia, thrombocytopenia, elevated lactate, and creatine elevation suggestive of acute kidney injury (Table 1). As a result, the patient met the criteria for sepsis, given the initial elevated temperature (>38°C), heart rate (>90 beats per minute), and white blood cell (WBC) count (>12 k/cumm).

**Table 1:** Pertinent laboratory values at initial presentation.

Laboratory test	Patient value	Reference range
White blood cell count (k/cumm)	13.5	4.5 - 10.0
Hemoglobin (g/dL)	11.9	13.5 - 16.5
Platelet count, auto (k/cumm)	57	160 - 360
Sodium (mmol/L)	135	135 - 145
Potassium (mmol/L)	4.3	3.5 - 5.1
Chloride (mmol/L)	101	100 - 110
Blood urea nitrogen (mg/dL)	24	8 - 22
Creatinine (mg/dL)	1.53	0.5 - 1.3
Glucose (mg/dL)	182	65 - 99
Lactate (mmol/L)	3.3	0.5 - 2.2

**Figure 1:**
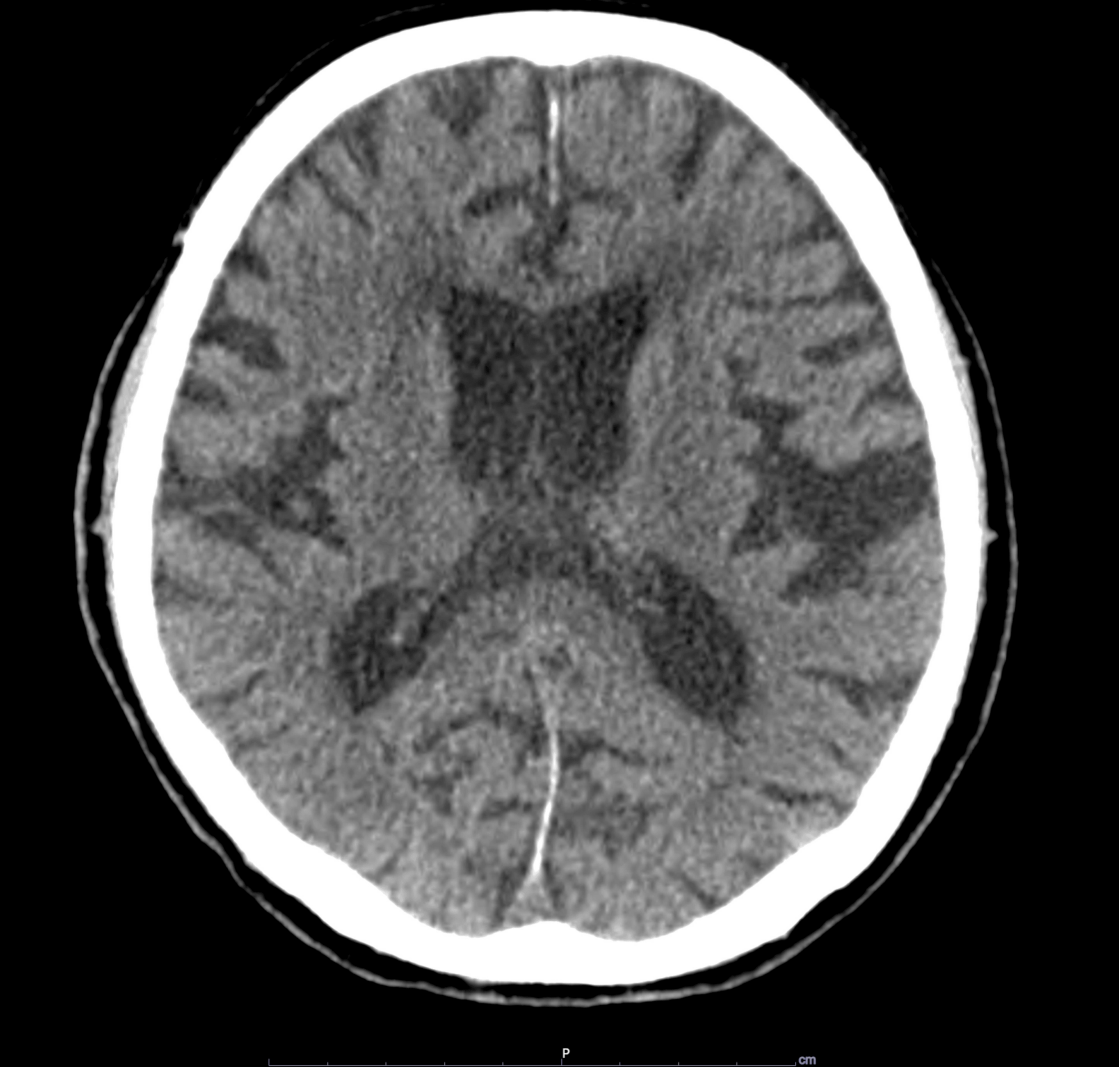
Cross-sectional view of computed tomography of the head without contrast.

Computed tomography (CT) of the head and cervical spine showed moderate white matter microvascular changes, with no intracranial hemorrhage (Figure 1). Chest X-ray, CT thoracic, and CT abdominal imaging did not show any acute processes. Unfortunately, an MRI of the brain was unable to be obtained, given the patient’s prior cochlear implants being MRI-incompatible. The patient was started on empiric ceftriaxone, vancomycin, acyclovir, and ampicillin, given high clinical suspicion for meningitis. An infectious disease specialist was consulted, who did not recommend adjunct steroid initiation. In the emergency department, lumbar puncture was attempted but was unsuccessful, and the patient was admitted for further workup.

The patient continued to be febrile, with new-onset rigors. The repeat exam showed new-onset right eye anisocoria. Repeat stat CT head imaging did not show any interim changes. HIV, rapid plasma reagin, and serum cryptococcal antigen were negative. Admission blood cultures obtained on day one grew two of four Gram-positive rods, later speciated to *L. monocytogenes*. Subsequent blood cultures and urine cultures on days two and three were negative. Successful lumbar puncture obtained on day three of hospitalization was notable for normal opening pressure, pleocytosis, low glucose, and high protein (Table 2). The pleocytosis showed a neutrophilic and lymphocytic split, 45% and 46%, respectively, which can be a classic hallmark sign of underlying listeriosis. Notably, the lumbar puncture was complicated by traumatic blood contamination of the specimen, as seen by the elevated cerebrospinal fluid (CSF) red blood cell count. The negative CSF cultures likely reflected antibiotic exposure prior to sample collection, lowering culture sensitivity. CSF meningoencephalitis (ME) panel was also performed. ME panels, often utilizing the BioFire FilmArray ME panel, are molecular multiplex polymerase chain reaction (PCR) tests designed to simultaneously detect 14 common bacterial, viral, and yeast pathogens. This returned positive hours later for *L. monocytogenes* on day three.

**Table 2:** Lumbar puncture cerebrospinal fluid (CSF) results. CSF: cerebrospinal fluid; ME: meningoencephalitis; PMN: polymorphonuclear neutrophils; PCR: polymerase chain reaction.

Laboratory test	Patient value	Reference range
CSF opening pressure (cm H_2_0)	18	10 - 20
CSF clarity	Slightly bloody	Clear
CSF red blood cell count (/cumm)	10,925	0 - 5
CSF cytology (/cumm)	211 (45% PMN, 46% lymphocytes, 8% monocytes, 1% basophils)	0 - 5
CSF glucose (mg/dL)	34	50 - 80
CSF protein (mg/dL)	407	15 - 45
CSF culture	Negative	Negative
CSF ME panel	*Listeria* *monocytogenes* PCR detected	Not detected

Subsequent antibiotic susceptibilities based on the initial positive blood cultures were also performed at reference laboratories (QuestLab), which showed *L. monocytogenes* to be susceptible to all antibiotics tested (Table 3). Therapy was streamlined to ampicillin monotherapy in accordance with antimicrobial stewardship principles favoring the narrowest effective spectrum. While meropenem possesses reliable anti-listerial activity, its broader spectrum offers no clear advantage in susceptible isolates and may increase selective pressure for antimicrobial resistance. Likewise, although aminoglycosides have traditionally been added for synergistic activity, available clinical data have not consistently demonstrated improved mortality or neurologic outcomes of aminoglycosides alone, whereas toxicity remains a concern. Therefore, per discussion with the infectious disease specialists, definitive treatment with ampicillin alone was selected following microbiologic confirmation and clinical improvement on hospital day three. The patient was treated for a total of three weeks’ duration from blood culture clearance, given *L. monocytogenes* bacteremia in the setting of *L. monocytogenes* meningitis. After one week of treatment, the patient demonstrated some cognitive improvement. He was now oriented to person and place, and intermittently following commands. He was ultimately discharged to a skilled nursing facility on hospital day 10 for continued physical and occupational therapy support.

**Table 3:** Antibiotic susceptibilities. MIC: minimum inhibitory concentration.

Antibiotic	Susceptibilities	MIC (μg/mL)
Ampicillin	0.5 – Susceptible	0.5
Meropenem	0.25 – Susceptible	0.25
Penicillin	0.5 – Susceptible	0.5
Trimethoprim-sulfamethoxazole	0.016 – Susceptible	0.016

## Discussion

*Listeria monocytogenes* is a Gram-positive bacterium generally transmitted through ingestion of contaminated food products [1]. Although exposure is widespread, invasive disease disproportionately affects immunocompromised individuals, with previously healthy hosts accounting for a minority of cases [7]. However, emerging epidemiologic data demonstrate that advanced age itself constitutes an independent risk factor for listeriosis, even in the absence of traditional immunosuppressive conditions [4,8]. Notably, the incidence of listeriosis rises sharply with advancing age, with individuals ≥85 years exhibiting an approximately 50-fold increased risk compared to younger adults [9].

Host defense against *L. monocytogenes *relies predominantly on cell-mediated immunity, particularly T-cell activation and macrophage-mediated intracellular killing [5]. Consequently, populations with impaired cellular immunity, including pregnant individuals, transplant recipients, diabetic patients, patients with HIV/AIDS, cirrhosis, chronic alcohol use, or those receiving immunosuppressive therapies, are at increased risk [3]. Importantly, aging is associated with a state of immunosenescence characterized by dysregulated cytokine signaling, reduced T-cell function, and impaired phagocytic activity, which may predispose elderly individuals to invasive listeriosis independent of comorbid disease [10]. Additional factors such as hypertension may further contribute by compromising blood-brain barrier integrity, potentially facilitating central nervous system invasion [11].

Clinically, invasive *L. monocytogenes* most commonly manifests as bacteremia or central nervous system infection, including meningitis and meningoencephalitis [12]. In elderly patients, presentation is frequently atypical, which can delay diagnosis and initiation of appropriate therapy. The classic triad of fever, neck stiffness, and altered mental status is observed less frequently in older adults, occurring in fewer than half of elderly patients, thereby reducing its diagnostic utility in this population [12].

An important teaching point highlighted by this case is the necessity of avoiding delays in antimicrobial therapy while simultaneously pursuing timely cerebrospinal fluid evaluation. Current guidelines recommend prompt lumbar puncture in suspected meningitis whenever safely feasible, with neuroimaging reserved for patients with clinical features concerning for elevated intracranial pressure or mass effect, including focal neurologic deficits, papilledema, new-onset seizures, severe immunocompromise, or markedly depressed consciousness [13]. When these features are present, CT imaging should precede lumbar puncture; however, antibiotic administration should not be delayed while awaiting imaging [13]. In our patient, early recognition and prompt lumbar puncture facilitated diagnosis and initiation of appropriate therapy.

Notably, in our case, the initial thrombocytopenia and acute kidney injury observed at presentation were most consistent with sepsis-associated organ dysfunction secondary to invasive listeriosis. Hematologic abnormalities, including thrombocytopenia, may occur in severe bacterial infections because of systemic inflammation, platelet consumption, and endothelial activation [14]. Likewise, acute kidney injury may be a common manifestation of sepsis resulting from a combination of renal hypoperfusion, inflammatory-mediated microvascular dysfunction, and direct cellular injury [14]. In our patient, both abnormalities improved during hospitalization with antimicrobial therapy and supportive care, paralleling the patient's clinical recovery.

Additionally, it is important to note that CSF culture may be negative while the CSF ME panel is positive for *L. monocytogenes*. This discordance has been documented in prior literature, as *L. monocytogenes *is notoriously difficult to detect by conventional methods [15]. As a facultative intracellular organism, *L. monocytogenes *may be sequestered within host cells, limiting recovery by standard culture techniques. It often also has low organism counts in CSF, reducing culture yield. Any prior antibiotic exposure reduces CSF culture sensitivity, while PCR-based methods can detect bacterial DNA even after antibiotics have rendered cultures negative [15]. As a result, CSF Gram stain may miss approximately two-thirds of *L. monocytogenes* meningitis cases, and CSF culture, while the gold standard for bacterial meningitis overall, has been shown to have reduced sensitivity for *L. monocytogenes* [15].

Despite advances in care, listeriosis remains associated with substantial mortality. The overall case fatality rate is estimated at approximately 20-30%, with significantly higher mortality observed in elderly patients and those with central nervous system involvement [12]. In the MONALISA study, authors were able to identify several independent predictors, including advanced age, active malignancy, multi-organ failure, decompensated comorbidities, monocytopenia, and elevated neutrophil count, with increased risk for mortality [16]. Furthermore, the presence of bacteremia in neurolisteriosis is associated with significantly worse outcomes, with lower survival rates compared to non-bacteremic cases [16]. Survivors may experience long-term neurologic sequelae, including progressive cognitive impairment, focal motor deficits, as well as an increase in malignancy-related mortality [17].

Early initiation of appropriate antimicrobial therapy is critical, as inadequate empiric treatment has been independently associated with increased 30-day mortality [18]. Notably, cephalosporins lack activity against *L. monocytogenes *and account for the majority of ineffective empiric regimens [11]. Current guidelines therefore recommend the inclusion of anti-listerial agents, such as ampicillin or penicillin, in empiric therapy for suspected bacterial meningitis in high-risk populations, including the elderly [11,18]. Consultation with infectious disease specialists or adherence to institutional guidelines is strongly recommended because management remains an area of ongoing debate.

Ampicillin remains the preferred first-line treatment for invasive listeriosis. Historically, combination therapy with gentamicin has been advocated because in vitro studies demonstrate synergistic bactericidal activity, and animal models have suggested enhanced intracellular killing [18,19]. However, clinical evidence supporting this practice is limited and largely derived from observational studies [19]. Several experts have questioned the routine use of aminoglycosides, given concerns regarding nephrotoxicity and ototoxicity, particularly among elderly patients and those with pre-existing renal dysfunction [19]. Some contemporary investigators argue that combination therapy is based on limited historical data and that high-dose beta-lactam monotherapy may be sufficient in many cases. Consequently, practice patterns vary among institutions, and decisions regarding adjunctive gentamicin should be individualized in consultation with infectious disease specialists.

Alternative antimicrobial options should also be considered when beta-lactams are contraindicated or intolerance develops. Trimethoprim-sulfamethoxazole (TMP-SMX) possesses excellent activity against *L. monocytogenes *and serves as the preferred alternative in patients with severe penicillin allergy [11,18]. In addition, some clinicians have utilized TMP-SMX in combination with ampicillin based on favorable intracellular penetration, although supporting evidence remains limited. Meropenem demonstrates reliable anti-listerial activity and may represent another acceptable alternative [19]. Fluoroquinolones, particularly levofloxacin and moxifloxacin, have exhibited in vitro activity and isolated clinical success, although experience remains limited, and these agents are generally considered salvage or secondary options rather than standard therapy [19].

The role of corticosteroids in *L. monocytogenes* meningitis remains controversial. Dexamethasone is recommended for suspected pneumococcal meningitis and is often administered empirically before pathogen identification [20]. However, once *L. monocytogenes* is identified, continuation of corticosteroids is debated. Some experts advocate discontinuation because the intracellular pathogenesis of *L. monocytogenes* differs from pneumococcal disease and because available evidence has not demonstrated a clear benefit [19]. Others argue that abrupt discontinuation after initial dosing is unlikely to be harmful. Given the absence of robust prospective data, decisions regarding continuation of dexamethasone should be individualized and ideally made in conjunction with infectious disease consultation [19].

Prevention through dietary counseling remains an important component of care. High-risk individuals, particularly elderly and immunocompromised patients, should be advised to avoid unpasteurized dairy products, soft cheeses made from unpasteurized milk, refrigerated deli meats unless reheated thoroughly, refrigerated smoked seafood, and other ready-to-eat foods associated with *L. monocytogenes c*ontamination [3]. Although no definitive source was identified in our patient, occult dietary exposure remains the most likely mechanism of infection and reinforces the importance of counseling regarding food safety practices.

## Conclusions

*Listeria monocytogenes* represents a significant and often underrecognized cause of invasive infection in the elderly population. Advanced age alone may potentially confer substantial susceptibility through immunosenescence-related mechanisms, even in the absence of traditional immunocompromising conditions. The frequently atypical clinical presentation, combined with high rates of morbidity and mortality, underscores the importance of maintaining a high index of suspicion in older adults presenting with suspected sepsis or meningitis. Prompt initiation of appropriate empiric antimicrobial therapy, including coverage for *L. monocytogenes*,is essential to improving outcomes. Greater awareness of age-related risk and tailored empiric treatment strategies are critical in optimizing care for this vulnerable population.
